# A Central Council

**Published:** 1892-06-25

**Authors:** 


### HOSPITAL OPINION.

[Contributions critical, suggestive, controversial, and technical are invited for this column, which is open to everyone who has anything to say that is of practical value and interest.]

### A CENTRAL COUNCIL.

Mr. Timothy Holmes, F.R.C.S., writes from 18, Great Cumberland Place, W.:—My attention has been called to an article in your journal for June 18th, headed "A Central Council," and signed "Agricola," in which the writer seems to suggest that in my speech at the annual meeting of the Charity Organization Society in April, I claimed some directing influence for that society in the new "Central Council" which we have reason to believe will be established if the recommendation of the Lords' Committee on Hospitals are adopted. The writer says that the society "is reckoning without its host in assuming that the chief places at the Central Council it desires to see created will be allotted to its nominees." Anyone who will read my speech will see that I said nothing of the kind, and I am sure I never thought of anything of the kind. The Charity Organization Society has made prolonged efforts, and successful efforts, to bring about the enquiry. It was to those efforts that I alluded, and not to the enquiry itself, as "a branch of the society's work." I should have thought no one could have so far misunderstood my plain language as to believe me capable of representing to an audience of reasonable men that the enquiry of the Lords' Committee was the work, and even *a branch* of the work, of the society. In that speech I gave full credit to those who formerly worked for the same good end, and of whom] I am proud to have been one. Their efforts, however, failed—those of the society which is according to this gentleman "regarded by the hospitals with a feeling stronger than suspicion" have succeeded; and I venture to say that the society has thereby rendered a service to the hospitals, the greatness of which will be more and more appreciated as time goes on. As to the other opprobrious terms which your correspondent is pleased to apply to the Charity Organization Society, I will content myself with stating that in my judgment, having known that society long, and having a very intimate knowledge of, and a very ardent attachment to, our hospital system, that system has no more intelligent and no more sympathetic supporters than are to be found in the ranks of the Charity Organization Society. If we knew who "Agricola" is, we should know better what right he has to pose as the representative of the hospitals, and to formulate their opinion about the society in question. For my own part, I have never thought myself entitled to "adopt," as he phrases it, "a representative attitude," though I have been connected with hospitals long enough and intimately enough to render me eager in advocating anything which seems to be for their advantage. Excuse my trespassing on your space. I would not have done so, but that it seemed probable that your readers might be misled as to the action of the society, of whose Council I was lately Chairman.

